# Supplementary material for: Identification of GB3 as a Novel Biomarker of Tumor-Derived Vasculature in Neuroblastoma Using a Stiffness-Based Model
Source: Cancers (Basel). 2024 Mar 5;16(5):1060. doi: 10.3390/cancers16051060 (PMC10931015; doi:10.3390/cancers16051060)
Supplement: Supplementary file 1 [file cancers-16-01060-s001.zip › cancers-2884253-supplementary.pdf]

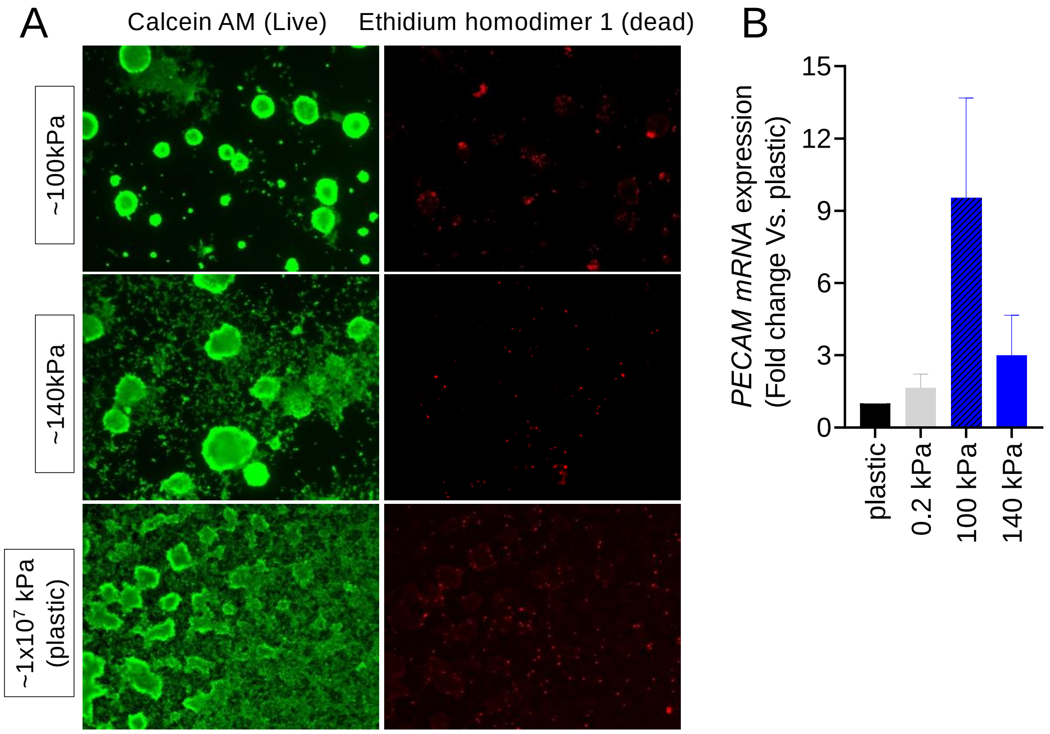

Figure S1. Studies of tumor-derived endothelial cells (TECs) cultured with RPMI and endothelial medium. (A) Live/dead cell viability assay of N-type neuroblastoma cells (SKN-BE(2)) cultured on the artery-liked PDMS substrates and plastic culture dishes. The cells were cultured in RPMI/ endothelial (EGM) culture medium for 7 days and then stained with the Calcein AM (live cells; green) and Ethidium homodimer 1 (dead cells, red). (B) PECAM mRNA levels in SKN-BE(2) cultured on the indicated substrates stiffness in RPMI/ endothelial (EGM) culture medium for 7 days. Fold change was calculated by first normalizing to GAPDH levels in the individual samples and then to the corresponding levels in cells cultured in plastic culture dishes. Data are shown as average ±SD (n=3).

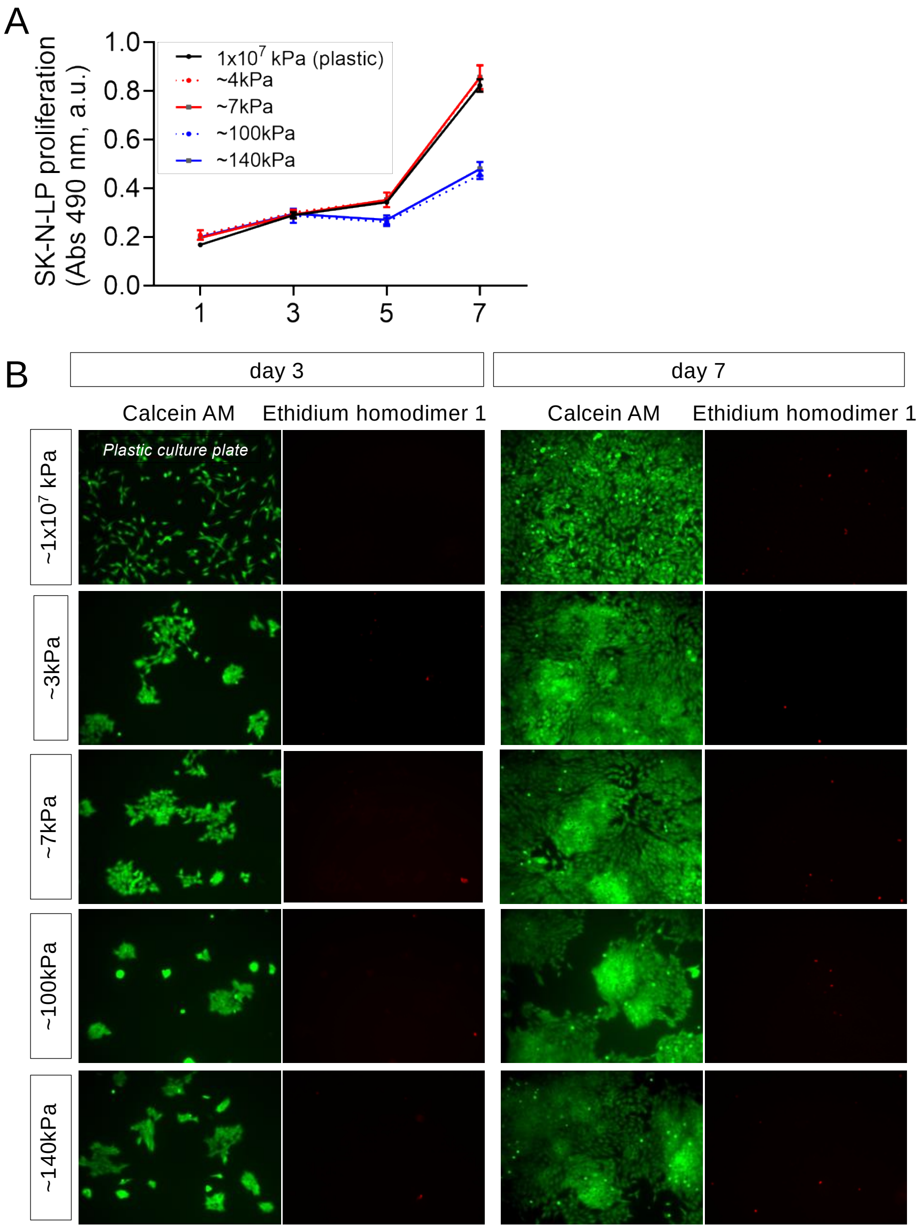

Figure S2. Proliferation and survival of I-type neuroblastoma on venous-like and artery-like PDMS materials. (A) Proliferation of I-type neuroblastoma cells (SK-N-LP) cultured on venous-like PDMS (red) and artery-like PDMS substrates (blue), compared to plastic dishes for the indicated time points (n=6). A colorimetric method was used (MTS assay). Absorbance (Abs) at 490nm (arbitrary units, a.u.) is proportional to cell number. (B) Live/dead fluorescence images of SK-N-LP cells cultured on the indicated substrates for 3 and 7 days (n=3). Calcein staining (green-live cells), ethidium homodimer-1 staining (red-dead cells).

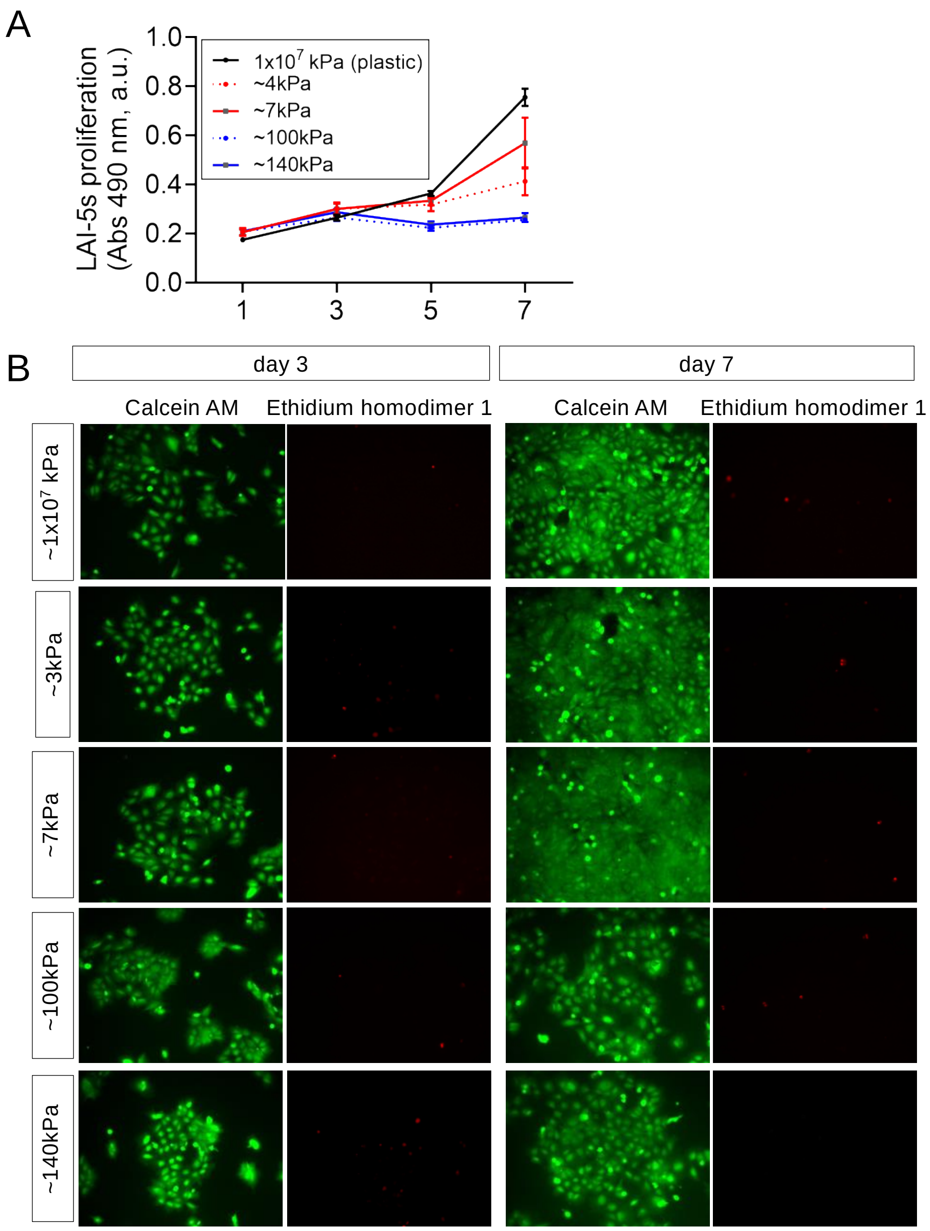

Figure S3. Proliferation and survival of S-type neuroblastoma cells LA1-5s on venous-like and artery-like PDMS materials. (A) Proliferation of S-type neuroblastoma cells (LA1-5s) cultured on venous-like PDMS (red) and artery-like PDMS substrates (blue), compared to plastic dishes for the indicated time points (n=6). A colorimetric method was used (MTS assay). Absorbance (Abs) at 490nm (arbitrary units, a.u.) is proportional to cell number. (B) Live/dead fluorescence images of LA1-5s cells cultured on the indicated substrates for 3 and 7 days (n=3). Calcein staining (green-live cells), ethidium homodimer-1 staining (red-dead cells).

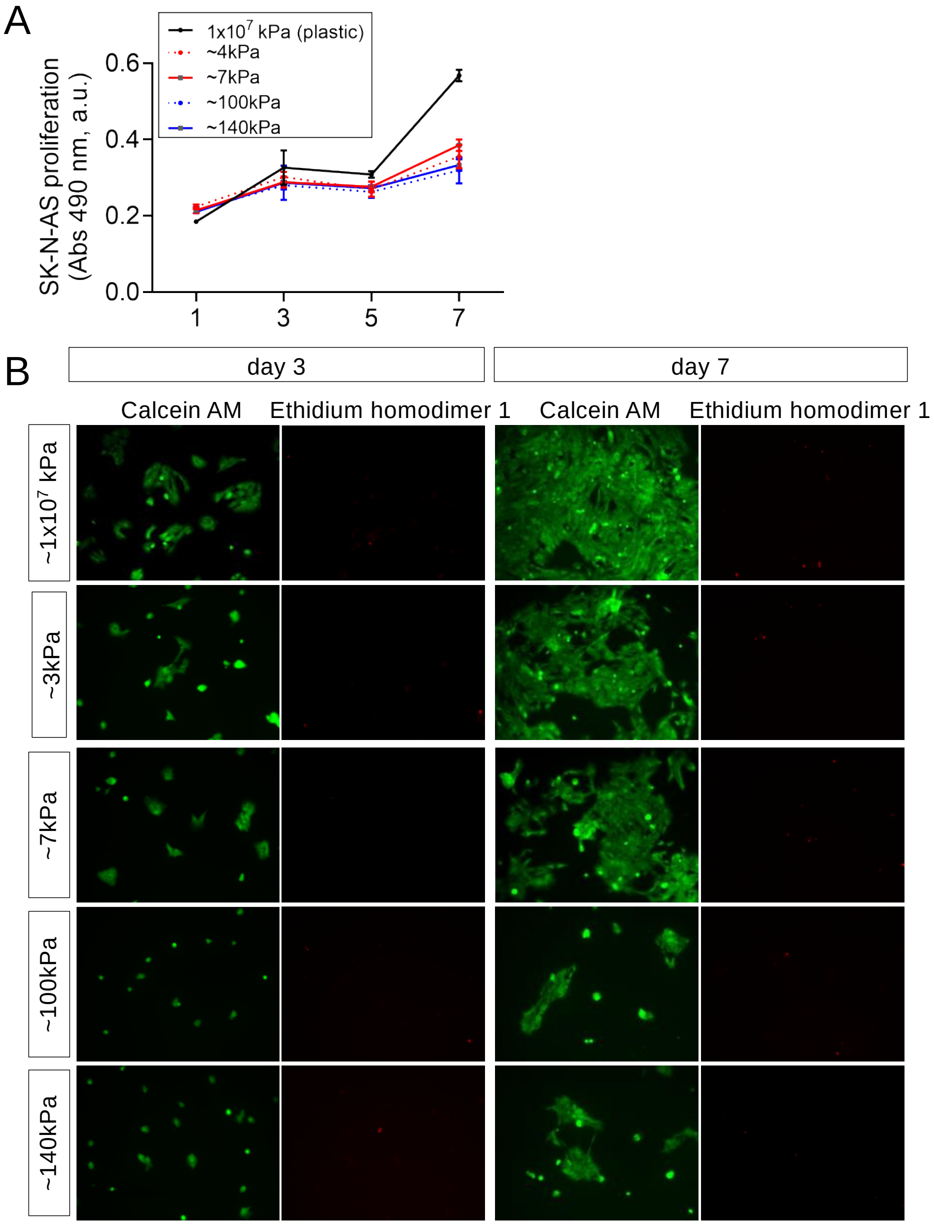

Figure S4. Proliferation and survival of S-type neuroblastoma cells SK-N-AS on venous-like and artery-like PDMS materials. (A) Proliferation of S-type neuroblastoma cells (SK-N-AS) cultured on venous-like PDMS (red) and artery-like PDMS substrates (blue), com-pared to plastic dishes for the indicated time points (n=6). A colorimetric method was used (MTS assay). Absorbance (Abs) at 490nm (arbitrary units, a.u.) is proportional to cell number. (B) Live/dead fluorescence images of SK-N-AS cells cultured on the indicated substrates for 3 and 7 days (n=3). Calcein staining (green-live cells), ethidium homodimer-1 staining (red-dead cells)
